# Community Synchrony in Aquatic Macroinvertebrates Is Unrelated to Environmental Variability but Differs Among Functional Feeding Groups

**DOI:** 10.1002/ece3.72999

**Published:** 2026-01-28

**Authors:** Anthony J. Pignatelli, Tad A. Dallas

**Affiliations:** ^1^ Department of Biological Sciences University of South Carolina Columbia South Carolina USA

**Keywords:** aquatic macroinvertebrates, environmental variability, functional groups, NEON, synchrony

## Abstract

Environments are becoming increasingly more variable, as a function of climate change. As this occurs, species may be exposed to conditions outside their preferred range. Such variability in the environment can influence community abundance as individual species respond either similarly (synchronous dynamics) or differently (asynchronous dynamics) to each other. These fluctuations in abundances are important for understanding the impact of environmental variability on species temporal fluctuations in aquatic macroinvertebrates. This group of organisms is species‐rich and highly sensitive to environmental fluctuations. We analyzed 18 stream macroinvertebrate communities sampled by the National Ecological Observatory Network between 2014 and 2022 to understand how community synchrony is related to stream temperature variability, discharge variability, and species turnover. We then quantified individual species contributions to community synchrony. These contributions were aggregated by functional feeding group to understand how resource acquisition strategies influenced species contributions. Species with higher contributions are often more synchronous with many other species. Here, community synchrony was expected to be negatively related to increasing environmental variability and turnover. Opposite our expectation, temperature variability, turnover, and discharge variability were unrelated to community synchrony. Contributions to community synchrony significantly varied among functional feeding groups. Scrapers had the highest proportion of taxa with significant positive contributions, followed by filterers. Shredders had the lowest proportion of species contributing to synchrony. Scrapers and shredders were significantly less synchronous than other functional feeding groups. This suggests that functional feeding group may explain patterns of community synchrony. Using a standardized, long‐term dataset, we demonstrated how temperature variability, turnover, and functional feeding groups relate to community synchrony. While identifying the drivers of community synchrony remains challenging, integrating functional groupings provides an approach to identify species that drive community dynamics.

## Graphical abstract

As environmental variability increases, as a function of climate change, communities will be less synchronous as species fluctuate more independently of each other. Species will contribute to community synchrony differently, based on their functional feeding groups.

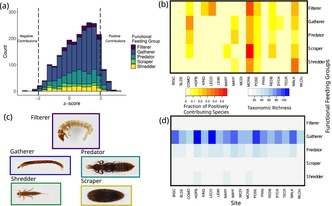

## 1 Introduction

A primary goal in community ecology is in understanding how species temporally fluctuate in response to various drivers (e.g., environment, species interactions). In multi‐species communities, species that fluctuate similarly in abundance over time are considered synchronous, whereas species that fluctuate differently from each other are asynchronous (Brown et al. 2016; Larsen et al. 2024; Loreau and de Mazancourt 2008). Community synchrony is commonly observed across many ecological systems and can offer insights into species dynamics such as the susceptibility of a community to an environmental perturbation (Brown et al. 2016; Larsen et al. 2024). Communities are rarely, if ever, completely synchronous or asynchronous but instead fall somewhere along this continuum. Although identifying the drivers of community synchrony remains challenging, evidence suggests that environmental stochasticity, interspecific competition, and species richness are considered some primary drivers, making it difficult to disentangle the effects of a particular driver (Ives et al. 1999; Loreau and De Mazancourt 2013; Valencia et al. 2020). In the presence of fluctuating environments, community synchrony patterns are unclear as environmental fluctuations can increase synchrony (Granzotti et al. 2024; Gu et al. 2023; Song et al. 2019) or decrease synchrony (Brown et al. 2016). Additionally, species‐rich communities are often less synchronous due to greater variation in species‐specific responses to environmental conditions as well as the influence of dominant species (Ives et al. 1999; Patrick et al. 2021; Valencia et al. 2020; Wang et al. 2019). Out of the many drivers of community synchrony, environmental variability is important, but the directional impact it has on communities remains unclear.

Local environmental conditions are becoming increasingly more variable, particularly through changes to temperature and precipitation, as a function of climate change (Burgmer et al. 2007; Harvey et al. 2020). For example, the frequency and magnitude of extreme heat and precipitation events are rapidly increasing (Harvey et al. 2020). These shifts in environmental variability have direct effects on population dynamics, altering community composition and relative abundance (Loreau and de Mazancourt 2008; Melbourne and Hastings 2008; Shoemaker et al. 2020, 2022). Additionally, increasing environmental variability may lead to species declines or extinctions, facilitate colonization by nonnative species, and alter overall community diversity through species turnover (Harvey et al. 2020; Jabot et al. 2020; Korhonen et al. 2010; Melbourne and Hastings 2008; Patrick et al. 2021; Rodriguez et al. 2025; Wilcox et al. 2017). Temporal species turnover could increase as species respond differently to these changes in environmental variability (Elmqvist et al. 2003; García‐Navas et al. 2021; Patrick et al. 2021; Wilcox et al. 2017). Species may differ in their responses to environmental variability depending on their physiology and behavior (Elmqvist et al. 2003), which can give rise to compensatory dynamics, where declines in the abundance of some species are offset by increases in others (Brown et al. 2016; Downing et al. 2014; Gonzalez and Loreau 2009; Jabot et al. 2020; Korhonen et al. 2010; Vasseur et al. 2005). In some cases, though, species may be similar enough at the taxonomic or functional scale that they are synchronous and, such compensation does not occur. Overall, compensatory dynamics have rarely been documented in natural systems (Barraquand et al. 2022). While theory predicts compensatory dynamics should occur due to environmental variation, the variability in temporal abundance among species has been found to generally decrease when in the presence of fluctuating environments, leading to more synchronous communities (Brown et al. 2016; Downing et al. 2014; Vasseur et al. 2005). Due to changing environmental conditions, evaluating the degree to which species fluctuate similarly or differently to each other may provide broader insights into community responses to environmental variability (Barraquand et al. 2022; Brown et al. 2016). Considering species' functional traits and groupings offers a complementary perspective for understanding the emergence of synchrony and asynchrony among communities.

We would expect that functionally similar species may fluctuate synchronously with each other and so incorporating the functional traits of species may help capture species‐specific differences that might lead to community asynchrony (García‐Navas et al. 2021; Granzotti et al. 2024; Siqueira et al. 2024; van Klink et al. 2019). For example, in ground beetle communities, greater interspecific variation in functional traits, such as body size and wing morphology, was associated with lower community synchrony (van Klink et al. 2019). One set of functional traits that influence species abundances includes functional feeding groups which may have impacts on the individual contribution of a species to community synchrony (Granzotti et al. 2024). Aquatic stream macroinvertebrates are classified into functional feeding groups that more specifically describe how organisms both acquire and consume resources. These groups include collector‐filterers, collector‐gatherers, shredders, scrapers, and predators, reflecting both the method of food acquisition and the specific type of resource consumed (Vannote et al. 1980; Wang et al. 2023). For example, filterers consume fine particulate organic matter suspended in the water column, scrapers graze periphytic algae found on rocks, and shredders process coarse organic material such as leaf litter (Vannote et al. 1980; Wang et al. 2023). The availability of these resources is closely tied to environmental conditions, particularly temperature and precipitation (Vannote et al. 1980; Walsh et al. 2005; Wang et al. 2023). Community synchrony has been explored across trophic levels where synchrony increases as trophic level increases (i.e., producer up to tertiary consumer), but because this looks across different taxa (e.g., plankton, fish, etc.), it ignores the relative role of mid‐level taxa and the specific functional feeding groups (i.e., macroinvertebrates) (Siqueira et al. 2024). Integrating functional traits with environmental variables may offer a more comprehensive explanation of community synchrony.

Here, we leveraged observational data of headwater stream macroinvertebrate communities from the National Ecological Observatory Network (NEON) to explore the relationship between community synchrony and environmental variability (here, stream temperature and discharge variability) and species turnover. Then we examined how community synchrony is influenced by species identity by quantifying species contributions to community synchrony and exploring whether contributions were explained by functional feeding groups. First (1), we expected that in communities experiencing higher environmental variability, species should fluctuate more asynchronously due to differences in species‐specific responses to environmental variability, leading to lower overall community synchrony. Second (2), communities experiencing higher species turnover would be less synchronous due to species replacement, the possible introduction of nonnative species, or the potential extinction of other species. Finally (3), species contributions to community synchrony will differ among functional feeding groups, where gatherers and filterers will contribute similarly while shredders, scrapers, and predators will have different and lower overall contributions. Overall, we found that community synchrony was not significantly related to environmental variability and species turnover, but significant differences in functional feeding group contributions to community synchrony were observed. Taken together, community synchrony is influenced by a complex suite of various biotic and abiotic drivers that are difficult to disentangle from observational data, but through the use of functional groups, species' traits may help to explain temporal fluctuations in the community.

## 2 Methods

### 2.1 Data and Field Collection Methods

**FIGURE 1:**
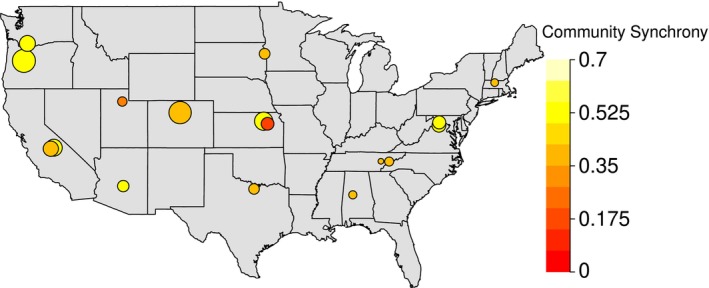
A map of the 18 field sites considered in this study. Each point represents the value of community synchrony observed at that particular site. Darker red colors indicate lower community synchrony (asynchrony) while lighter yellow colors indicate higher community synchrony. Point sizes represent taxonomic richness at the site where larger points indicate higher taxonomic richness while smaller points indicate lower taxonomic richness.

Data were obtained from the National Ecological Observatory Network using the neonUtilities package in R (NEON; Lunch et al. 2023; R Core Team 2021), specifically macroinvertebrate community abundance (DP1.20120.001), stream discharge (DP1.20048.001), and stream temperature (DP1.20053.001). Macroinvertebrate communities were sampled from 18 small streams classified by NEON as wadeable stream reaches accessible throughout most of the year. Sampling was conducted along a 1 km reach at each site to capture multiple habitat types. Macroinvertebrate sampling was standardized across NEON sites for data comparability, where each site was sampled three times per year (spring, summer, and fall). At each sampling event, collections were taken from the two most dominant habitat types (e.g., riffles, runs, pools) based on visual assessment. Taxa were identified to genus or species level; however, analyses were conducted at the genus level to ensure consistent taxonomic resolution across sites (NEON 2024). Prior to analysis, population density was calculated for each species to account for sampling effort in the NEON data. There was a wide range of taxonomic richness across the 18 sites (109 to 228 genera, with a mean of 158; see Supporting Information). We considered all 18 sites in the contiguous United States, from 2014 to 2022 (Figure 1; Table 1). All analyses were conducted in R (v. 4.3.3, R Core Team 2021).

**TABLE 1:** Site level values for taxonomic richness (S), average temperature variability (Temp), average temporal species turnover, average discharge variability (Discharge), community synchrony (ϕ), and the associated p‐value for synchrony (from Monte Carlo randomizations). If community synchrony is significant, this means that the community is more synchronous compared to the null randomizations.

Site	Lat.	Long.	S	Temp (°C)	Turnover	Discharge ($m^{3}/s$)	ϕ	p
MAYF	32.96	−87.41	179	18.51	0.42	0.015	0.16	< 0.001
SYCA	33.75	−111.51	134	14.75	0.63	0.438	0.43	< 0.001
TECR	36.96	−119.03	148	1.22	0.24	0.005	0.03	0.386
BIGC	37.06	−119.26	174	13.52	0.32	0.005	0.21	< 0.001
COMO	40.03	−105.54	126	3.47	0.25	0.015	0.18	< 0.001
WLOU	39.89	−105.92	109	2.16	0.23	0.015	0.24	< 0.001
KING	39.11	−96.60	123	10.24	0.63	0.006	0.22	< 0.001
MCDI	38.95	−96.44	126	10.49	0.48	0.007	0.19	< 0.001
HOPB	42.47	−72.33	223	11.17	0.29	0.015	0.18	< 0.001
MCRA	44.26	−122.17	157	5.65	0.23	0.016	0.62	< 0.001
LECO	35.69	−83.50	195	13.29	0.28	0.016	0.08	< 0.001
WALK	35.96	−84.28	186	12.87	0.21	0.145	0.10	< 0.001
PRIN	33.38	−97.78	164	16.13	0.34	0.004	0.01	0.674
REDB	40.78	−111.80	142	8.57	0.27	0.098	0.29	< 0.001
LEWI	39.10	−77.98	132	13.33	0.31	0.006	0.20	0.003
POSE	38.89	−78.15	228	11.28	0.27	0.002	0.10	< 0.001
MART	45.79	−121.93	159	9.23	0.22	0.021	0.16	< 0.001
BLDE	44.95	−110.59	143	1.32	0.21	0.060	0.26	< 0.001

### 2.2 Community Synchrony

Community synchrony was estimated for each site as a single value for the full time series. Synchrony was calculated following the approach proposed by Loreau and de Mazancourt (2008) (Equation (1)), which estimates community synchrony as the ratio of overall community abundance variance ($\sigma_{\mathrm{xT}}^{2}$) to the squared sum of species‐level standard deviations ${\left(\sum_{i}\sigma_{x}\right)}^{2}$ with a single value obtained bounded between zero (0) and one (1). Here, a value of zero indicates fully asynchronous fluctuations in species abundance while a value of one is when species are fluctuating fully synchronously (Loreau and de Mazancourt 2008) (Equation 1).

(1) $$\phi_{x}=\frac{\sigma_{\mathrm{xT}}^{2}}{{\left(\sum_{i}\sigma_{x}\right)}^{2}}$$

Because sampling occurred three times per year over short periods, we averaged population densities across sampling events within each year to generate annual density estimates for each taxon before calculating community synchrony. By averaging population density across the three time points, we can better capture long‐term abundance trends for each taxon. Community synchrony was calculated using the community.sync function in the synchrony package. The value of observed community synchrony is then compared to a null model (*α* = 0.05) where community synchrony is estimated by 999 Monte Carlo randomizations of the community abundance matrix (Gouhier and Guichard 2014).

### 2.3 Environmental Variability & Taxonomic Turnover

Here, environmental variability consisted of stream temperature variability and discharge variability, both of which have demonstrated effects on aquatic macroinvertebrate abundance (Death 2008; Hette‐Tronquart et al. 2013; Larsen et al. 2024; Walsh et al. 2005). Variability in temperature and discharge was quantified as the standard deviation of the full time series at each site. Species turnover was calculated temporally at the genus level for each year at each site as the proportion of taxa either gained or lost relative to the total number of taxa observed across a time period within a single site, using the codyn package (Hallett et al. 2016). We explored site‐level trends in temporal turnover in the Supporting Information (Figure S3). Spearman's rank correlations (*α* = 0.05) were conducted to test for relationships between community synchrony and each environmental variable (temperature variability, discharge variability) and species turnover. Because species richness and community diversity have been found to have a relationship with community synchrony (Loreau and de Mazancourt 2008; Valencia et al. 2020), we also explored whether species richness and Simpson diversity were related to community synchrony in the Supporting Information. Mean richness and diversity were calculated for each site using the vegan package (Oksanen et al. 2022), and Spearman's rank correlations were conducted with community synchrony (see Supporting Information, Figure S2). Although communities were taxonomically rich and diverse, both were unrelated to community synchrony (see Supporting Information, Figure S2). Finally, to identify if community synchrony was similar across geographic space, we tested for spatial autocorrelation in environmental variability, turnover, and community synchrony using Moran's *I*, finding no evidence of spatial autocorrelation (see Supporting Information, Table S1).

### 2.4 Species and Functional Feeding Group Contributions to Synchrony

We estimated each taxon's contribution to community synchrony, relative to a null distribution, by calculating a z‐score based on randomization procedures. For each taxon, its abundance time series (i.e., its column in the community matrix) was randomized 100 times while keeping all other taxa unaltered. Community synchrony was recalculated after each randomization to generate a null distribution of community synchrony for that specific taxon. The observed synchrony value was then compared to this null distribution to calculate a z‐score, representing the standardized difference between observed and expected synchrony for that taxon. This procedure was repeated for all taxa, resulting in an individual z‐score for every taxon in the community. Taxa with significant positive z‐scores (*z* > 1.96, *α* = 0.05) were interpreted as contributing positively to synchrony, indicating that they fluctuated similarly with many other taxa. Taxa with significant negative z‐scores (*z* < −1.96, *α* = 0.05) were interpreted as contributing to asynchrony, indicating that their fluctuations were independent of the broader community.

To examine functional feeding group‐level patterns, taxa were assigned to functional feeding groups using taxonomic information from *The Atlas of Common Freshwater Macroinvertebrates of Eastern North America* (Morse et al. 2020). Taxa were assigned to one of five functional feeding groups: filterer, gatherer, scraper, shredder, or predator. Taxa were then aggregated by functional feeding group, yielding a distribution of z‐scores for each group. We calculated the proportion of taxa in each functional feeding group that had a significant positive or negative contribution to community synchrony. To account for taxa present across multiple sites, contributions by functional feeding group were analyzed using a linear mixed effects model where the z‐scores were the response, sites and genera were random effects, and functional feeding group was the fixed effect.

## 3 Results

### 3.1 Environmental Variability

**FIGURE 2:**
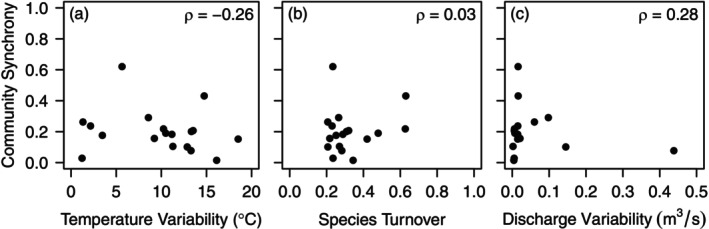
Community synchrony was not significantly related (*p* > 0.05) to temperature variability, species turnover, or discharge variability. Communities were relatively similar in the amount of discharge variability and turnover experienced. Sites differed in the amount of temperature variability they experienced. While non‐significant, each panel has its associated Spearman's correlation coefficient (ρ).

Across sites, temperature variability often exceeded 10°C, ranging from 1°C to 18°C. Species turnover ranged from about 0.2 to 0.6, but most sites experienced average annual turnover of about 0.3 (Figure 2a,b). We explored the relationship between temperature variability and turnover, finding that there was a positive correlation (see Supporting Information, Figure S1). Discharge variability was low across most sites, with 17 of the 18 sites having a discharge variability of less than 0.2 m^3^/s. The site with the highest discharge variability (0.43 m^3^/s) was a desert stream in Arizona that often experiences flash floods. Across sites, mean stream discharge variability was 0.05 m^3^/s. Most sites exhibited low community synchrony with only one site having a community synchrony value of 0.62 (Table 1). While most communities were significantly asynchronous (Table 1), community synchrony was not significantly related to stream temperature variability (*ρ* = −0.26, *p* = 0.29, Figure 2a), temporal species turnover (*ρ* = 0.03, *p* = 0.91, Figure 2b), or stream discharge variability (ρ = 0.28, *p* = 0.25, Figure 2c).

### 3.2 Species Contributions to Synchrony

**FIGURE 3:**
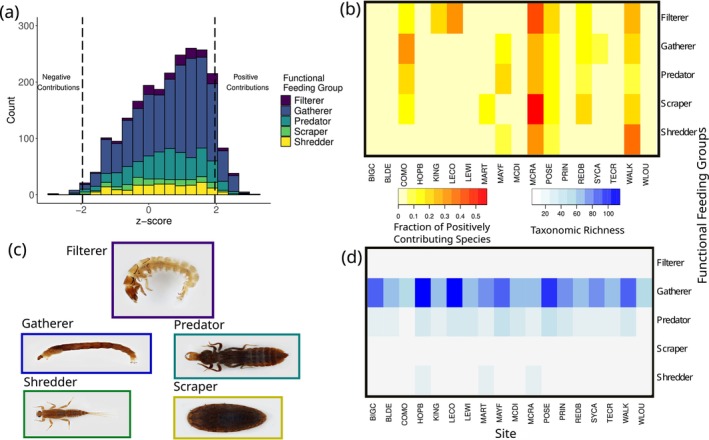
Summary of the functional feeding group analysis. (a) A stacked histogram showing the distribution of z‐scores for each functional feeding group which indicate positive or negative contributions to community synchrony. Vertical dashed lines represent the significance level at which significant contributions were interpreted (*α* = 0.05). (b) A heat map showing the proportion of taxa with significant positive contributions to community synchrony by functional feeding group across sites. Darker red boxes indicate a higher proportion of positively contributing taxa. (c) Example images of taxa found in each functional feeding group. Images provided by The Atlas of Common Freshwater Macroinvertebrates of Eastern North America (Morse et al. 2020). (d) A heat map showing the number of taxa for each functional feeding group that were used to estimate functional feeding group contributions to community synchrony. Gatherers had a higher number of taxa than all other functional feeding groups, but does not translate into having the highest positive contribution to community synchrony.

**FIGURE 4:**
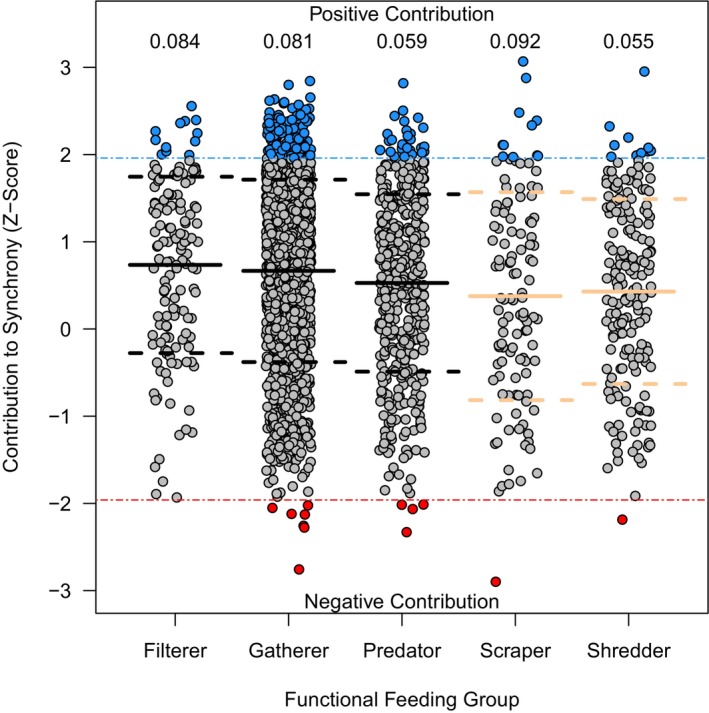
Raw z‐scores for genera across sites for the five functional feeding groups. Points colored blue indicate significant positive contributions to community synchrony and red points indicate significant negative contributions to synchrony. Positive contributing taxa were contributing to synchronous fluctuations while negative contributing taxa contributed to asynchrony. Overall, most taxa did not have a significant contribution to community synchrony as evidenced by the large number of gray points. Values above each column are the proportion of taxa with significant positive contributions to community synchrony for the specific functional feeding group. Solid horizontal lines at each group show the average contribution to synchrony and the associated dashed lines represent the standard deviation of the group. Horizontal lines of the same color indicate that those functional groups were more synchronous with each other.

Differences in species‐level contributions to community synchrony were observed across taxa. In a single site (MCRA), all five functional feeding groups had more than 30% of taxa positively contribution to synchrony (Figure 3b). This site also had the highest observed community synchrony value (ϕ = 0.62, Table 1). Functional feeding group taxonomic richness did not exhibit a clear pattern (Figure 3d). Overall, each functional feeding group had less than 10% of taxa with significant positive contributions to community synchrony (Figure 4). Filterers were the only group that had no taxa negatively contributing to community synchrony. Scrapers had the highest proportion of taxa with positive contributions to synchrony (9.2%) while shredders had the lowest (5.5%) (Figure 4). Overall, significant differences in contributions to community synchrony were observed among functional feeding groups. After accounting for site and genus‐level differences, scrapers (*t* = −2.44, *p* < 0.05) and shredders (*t* = −2.29, *p* < 0.05) strongly contributed to community asynchrony and were overall less synchronous with filterers, gatherers, and predators (Figure 4).

## 4 Discussion

We failed to detect a significant relationship between community synchrony and environmental variability as well as species turnover in stream macroinvertebrate communities. However, differences were observed in how species contributed to overall community synchrony based on functional feeding group identity. This highlights the challenge of disentangling the drivers of community synchrony, while demonstrating the utility of functional groups as a way to explain how individual species are contributing to overall community synchrony. Our findings emphasize the need to identify and consider additional potential drivers of community dynamics, such as functional traits, to gain a more comprehensive understanding of the mechanisms underlying synchrony.

While we found no relationship between community synchrony and environmental variability, overall community synchrony was low, reinforcing the complexity of attributing synchrony to environmental variability (Vasseur et al. 2005). Because insects are particularly susceptible to changes in thermal conditions (González‐Tokman et al. 2020; McNamara et al. 2021), we expected to observe a negative relationship between environmental variability and community synchrony, and an absence of a relationship may indicate that other factors not explicitly captured may be affecting species fluctuations in these communities. The magnitude of environmental variability did not greatly influence community synchrony, such that communities experiencing low temperature variability were similar in community synchrony values to communities experiencing higher temperature variability. Headwater streams can be fed by different sources, such as from seasonal snow melt or persistent groundwater extrusion, which can impact water temperatures (Brooks et al. 2025; Durance and Ormerod 2009). Relatedly, discharge variability could have been impacted by the frequency of precipitation events, something not considered here (Durance and Ormerod 2009). The NEON sampling protocol explicitly reserves two discharge sampling events for high flow events, and so our discharge variability estimates do include possible seasonal bouts of high flow (Lunch et al. 2023). Headwater streams may generally experience strong seasonality in discharge, which may be masked by our single value estimations of discharge variability, which covered an 8‐year time period (Death 2008; Durance and Ormerod 2009). Even so, due to the wide distribution of NEON sites, the discharge measurements are a broad generalization of other streams in natural areas across the US. Overall, we suggest that environmental variability alone is not responsible for the asynchrony observed in these communities (Brown et al. 2016; Downing et al. 2014).

While species turnover was not related to community synchrony, the communities overall experienced high rates of turnover while being relatively asynchronous (Baranov et al. 2020; Brown et al. 2016). The high rates of turnover could be explained by a number of things such as species extinction, re‐colonization, or species invasion (Baranov et al. 2020; Brown et al. 2016). An increase in average temperature and variability in temperature ranges may allow for new species to colonize, thus increasing rates of turnover (Baranov et al. 2020; McNamara et al. 2021). This may be the case as environmental conditions become favorable to species that did not previously occur at a given site and less tolerable to existing species in the community (Baranov et al. 2020; McNamara et al. 2021). The role of invasive species in these communities was not considered, but theoretical work has shown that asynchronous communities may be more susceptible to species invasion (Davidson and Shoemaker 2023). From what was observed, turnover does not seem to have a direct relationship with community synchrony.

In the case of aquatic macroinvertebrates, functional feeding groups were shown to differ in how they contributed to community synchrony. Food resources for aquatic macroinvertebrates are strongly influenced by environmental conditions, and so species may be tracking changes in their environment through food availability which we were not able to examine using observational data (Doretto et al. 2020; Vannote et al. 1980). While quantifying the contribution of individual taxa to community synchrony can be informative, it is challenging to interpret in species‐rich communities. Grouping species by something such as functional feeding groups provides a more tractable method for identifying which traits and specifically which types of species have a greater influence on community synchrony (Granzotti et al. 2024; Viviani et al. 2019). Although gatherers were more widely observed, this did not translate into more positive contributions to community synchrony. For many taxonomic groups, increasing richness may promote asynchronous fluctuations within the community, as has been observed in other systems (De Mazancourt et al. 2013), but in the case of these communities, there was no observable pattern between richness and contributions to synchrony. Filterers, for example, are not species rich but are constantly present in the community over time as they are less reliant on seasonal food resources, thus exhibiting a more homogeneous distribution throughout the stream (Jacquet et al. 2022; Wang et al. 2023). Shredders and scrapers also exhibited lower taxonomic richness, but scrapers, specifically, contained more positively contributing species. These taxa are generally more common in headwater streams, and respond to seasonal variation in resource availability more strongly than other taxa (Walsh et al. 2005; Wang et al. 2023). Shredders, for example, rely on leaf litter as a food source, and so they may be more abundant in the autumn compared to spring or summer which could contribute to asynchrony with species from different functional feeding groups (Wang et al. 2023). These observed differences among functional feeding groups demonstrate that the inclusion of functional traits may help to better understand patterns of community synchrony (García‐Navas et al. 2021; van Klink et al. 2019).

Community synchrony is an estimate for a single trophic level; thus, it overlooks potential interactions from species in higher or lower trophic levels. While our consideration of functional feeding groups included macroinvertebrate predators, we did not explore the role of primary producers found in lower trophic levels or the impact of larger predators such as fish. Food web structure may be an important contributor to community synchrony (Danet et al. 2021). Alterations to food web structure, as a function of temperature variability, have been observed in stream macroinvertebrate communities (Hette‐Tronquart et al. 2013). Collector‐filterers and shredders were attributed to explaining differences in food web structure among some temperate streams (Hette‐Tronquart et al. 2013). Understanding the structure of food webs may reveal how interactions between trophic levels, from top predators to primary producers, shape patterns of community synchrony.

Community synchrony can be driven by a range of abiotic (e.g., environment) and biotic (e.g., functional traits/groups) factors rather than a singular driver, making it difficult to disentangle these drivers from observational data alone. By considering species' functional feeding groups, we gained more information into how species might be contributing to overall community synchrony. The low sample size (18 sites) and coarse temporal resolution (3 samples per year for a site) may have been a factor in not detecting a relationship, but utilizing known species functional traits and functional groups may prove more useful in identifying factors leading to community synchrony than the environment alone. Identifying whether communities are synchronous or asynchronous may be useful in conservation management strategies as certain species can be targeted based on how they are temporally fluctuating with other species. Specifically considering functional traits and groupings can help with ensuring high functional diversity in a community. In a constantly changing world, understanding what leads to communities becoming synchronous or asynchronous will be important for future community stability and biodiversity in the face of climate change.

## Author Contributions

**Tad A. Dallas:** conceptualization (equal), formal analysis (supporting), funding acquisition (lead), methodology (lead), resources (lead), supervision (lead), writing – review and editing (equal). **Anthony J. Pignatelli:** conceptualization (equal), data curation (lead), formal analysis (lead), visualization (equal), writing – original draft (lead), writing – review and editing (lead).

## Funding

This work was supported by grants from the National Science Foundation (NSF‐DEB‐2420769 and NSF‐DEB‐2017826) awarded to Tad Dallas.

## Conflicts of Interest

The authors declare no conflicts of interest.

## Supporting information


**Data S1:** ece372999‐sup‐0001‐Supinfo.pdf.

## Data Availability

R code and data files are available on figshare at https://figshare.com/s/7a6aa97fe73e44b8c19e.
