# Supplementary material for: Community Synchrony in Aquatic Macroinvertebrates Is Unrelated to Environmental Variability but Differs Among Functional Feeding Groups
Source: Ecol Evol. 2026 Jan 28;16(2):e72999. doi: 10.1002/ece3.72999 (PMC12852502; doi:10.1002/ece3.72999)
Supplement: Supplementary file 1 — Data S1: ece372999‐sup‐0001‐Supinfo.pdf. [file ECE3-16-e72999-s001.pdf]

Supporting Information

Community synchrony is negatively related to temperature variability in freshwater macroinvertebrates

Relationship between Temperature Variability and Turnover We explored the possibility that temperature variability covaried with taxonomic turnover. Here, temperature variability and turnover values from the main text were used. We conducted a Spearman's rank correlation between temperature variability and taxonomic turnover across all sites and found that there was a significant positive relationship between them (ρ = 0.66, p = 0.004, Fig. S1). Even with the significant correlation between temperature variability and turnover, we could not assume that the two parameters were interacting in any way, thus, we considered their relationship to community synchrony independently in our main text analysis.

Community Composition For each site, we calculated species richness as the total number of unique species and community diversity was calculated as Simpson Diversity, using the `vegan` package (Oksanen et al., 2022). The relationship between species richness and community diversity was observed with community synchrony using Spearman correlations. There was a non-significant negative correlation between species richness and community synchrony (ρ = -0.177, p = 0.48, Fig. S2a). Species richness ranged from 109 in WLOU to 228 in POSE with mean species richness of 158 across all sites. Additionally, there was a non-significant positive correlation between Simpson's diversity and community synchrony (ρ = -0.065, p = 0.7986, Fig. S2b). The highest diversity was 0.905 at HOPB and SYCA had the lowest diversity at 0.721, but overall, diversity was high across all sites.

Species Turnover These communities are highly speciose and while average turnover was high across sites, we visually explored the timeseries of species turnover for each site. Overall, sites experienced limited fluctuations in turnover over time (Fig. S3).

Moran's I Spatial Autocorrelation Spatial autocorrelation was calculated for stream temperature variability, discharge variability, species turnover, and community synchrony using the `Moran.I` function within the `ape` package (Paradis and Schliep 2019). There was no signif-

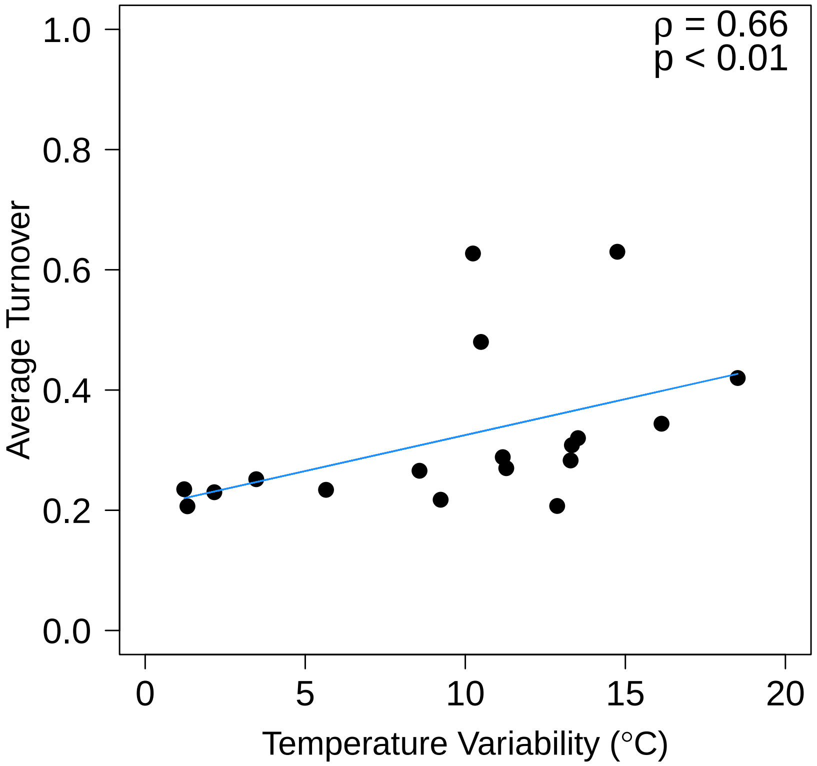

Figure S1: There was an observed significant positive relationship between temperature variability and turnover whereas increasing temperature variability often was associated with increasing rates of turnover (ρ = 0.66, p < 0.01). The solid blue line is for visualization purposes and does not represent a model fit.

icant spatial autocorrelation in temperature variability (p = 0.524), discharge variability (p = 0.608), or turnover (p = 0.382, Table. S1). Additionally, community synchrony was not spatial autocorrelated across sites (p = 0.364, Table. S1).

References

Paradis, E., and K. Schliep. 2019. "Ape 5.0: An Environment for Modern Phylogenetics and Evolutionary Analyses in R." Bioinformatics 35:526-528.

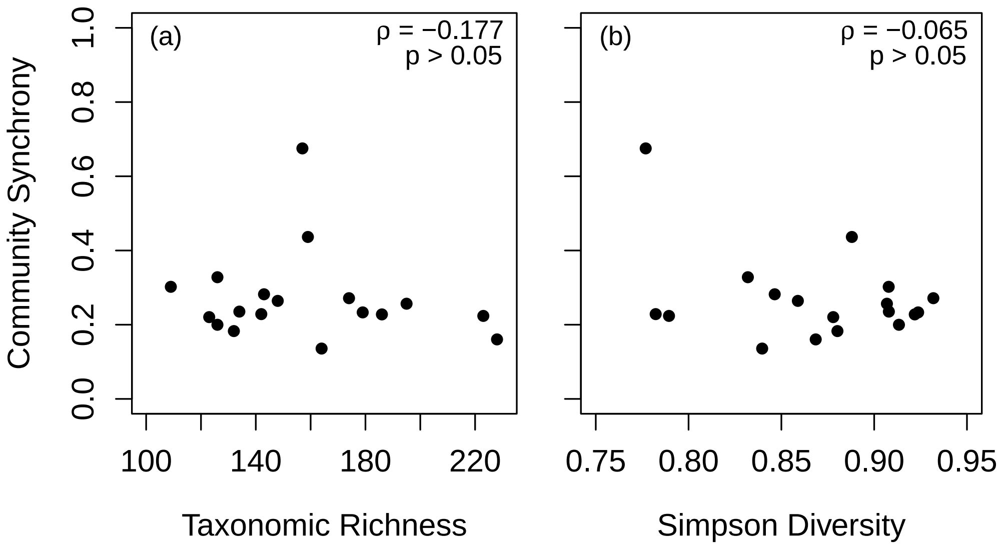

Figure S2: Species richness did not have a significant relationship with community synchrony (panel a, ρ = -0.177, $p > 0.05$). Similarly, there was not a significant relationship between community diversity and community synchrony (panel b, ρ = -0.065, $p > 0.05$).

Table S1: Statistical results from Moran's analysis for environmental variability, species turnover, and community synchrony. Expected values are the Moran's I value expected under the null hypothesis. Observed values are the actual computed Moran's I of the real data. The SD values are the standard deviation of the Moran's I under the null. The data did not display spatial autocorrelation.

Variable	Expected	Observed	SD	p-value
Temperature Variability	-0.05882353	-0.03436304	0.0384	0.524
Discharge Variability	-0.05882353	-0.04416218	0.0286	0.608
Species Turnover	-0.05882353	-0.02657737	0.0369	0.382
Community Synchrony	-0.05882353	-0.02895678	0.0329	0.364

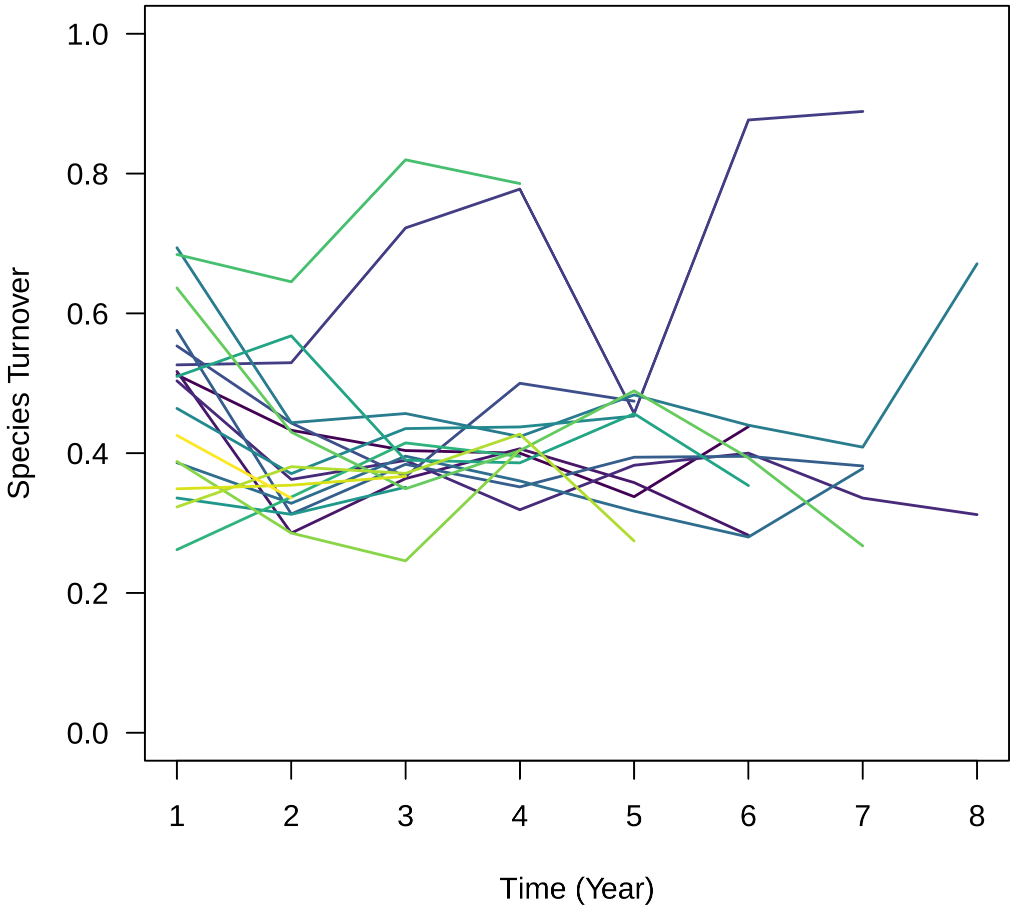

Figure S3: Across all sites, species turnover was relatively consistent over time. All sites experienced turnover above 0.2, while a single site experienced a high turnover above 0.8. This shows that these communities are highly dynamic and variable over time.
